# A new CUSUM control chart under uncertainty with applications in petroleum and meteorology

**DOI:** 10.1371/journal.pone.0246185

**Published:** 2021-02-04

**Authors:** Muhammad Aslam, Ambreen Shafqat, Mohammed Albassam, Jean-Claude Malela-Majika, Sandile C. Shongwe

**Affiliations:** 1 Department of Statistics, Faculty of Science, King Abdulaziz University, Jeddah, Saudi Arabia; 2 Department of Statistics and Financial Mathematics, Nanjing University of Science and Technology, Nanjing, Jiangsu, P.R. China; 3 Department of Statistics, Faculty of Natural and Agricultural Sciences, University of Pretoria, Hatfield, South Africa; 4 Department of Statistics, College of Science, Engineering and Technology, University of South Africa, Pretoria, South Africa; University of Defence in Belgrade, SERBIA

## Abstract

In these last few decades, control charts have received a growing interest because of the important role they play by improving the quality of the products and services in industrial and non-industrial environments. Most of the existing control charts are based on the assumption of certainty and accuracy. However, in real-life applications, such as weather forecasting and stock prices, operators are not always certain about the accuracy of an observed data. To efficiently monitor such processes, this paper proposes a new cumulative sum (CUSUM) $\bar{X}$ chart under the assumption of uncertainty using the neutrosophic statistic (NS). The performance of the new chart is investigated in terms of the neutrosophic run length properties using the Monte Carlo simulations approach. The efficiency of the proposed neutrosophic CUSUM (NCUSUM) $\bar{X}$ chart is also compared to the one of the classical CUSUM $\bar{X}$ chart. It is observed that the NCUSUM $\bar{X}$ chart has very interesting properties compared to the classical CUSUM $\bar{X}$ chart. The application and implementation of the NCUSUM $\bar{X}$ chart are provided using simulated, petroleum and meteorological data.

## Introduction

Control charts play an essential role in monitoring processes in the production and manufacturing sectors. Shewhart-type control charts are the most popular charts because of its simplicity and attractive sensitivity towards large shifts in the process parameters. These charts are widely applied in many fields and operations (such as manufacturing processes, health care monitoring, stock exchange, credit card, financial fraud detection, weather updates, and internet traffic flow, etc.). However, Shewhart-type charts are inefficient in detecting small to moderate process shifts. Due to this drawback, researchers have developed new control charts based on classical and adaptive sampling designs to detect small-to-moderate process shifts as quickly as possible, including large shifts. Control charts are classified into memory-type (e.g. cumulative sum (CUSUM), exponentially weighted moving average (EWMA), etc.) and memoryless (e.g. Shewhart-type) control charts; see for instance [1]. The latter uses only the latest information of the process to compute the charting statistics, which makes it less sensitive to detect small-to-moderate shifts, but enhances its ability to detect large shifts in the process. The first (i.e. memory-type control chart) uses both current and past information to compute the charting statistics, which makes it more sensitive in detecting small-to-moderate shifts and less sensitive in detecting large shifts in the process. The CUSUM and EWMA charts are the most popular memory-type control charts. To enhance the aforementioned charts, various advanced techniques are used including the addition of run rules, the use of variable sampling interval (VSI) and variable sample size (VSS) sampling designs for adaptive charts. Among all the advanced developments in the statistical process control (SPC) field, the CUSUM chart is an efficient alternative to the Shewhart chart when it comes to the detection of small to moderate shifts, see [1].

Both memory-type and memory-less control charts are used to monitor and control the spread or location parameters in any process. The sensitivity of memory-type charts have attracted the attention of many researchers and brought many more improvements in the SPC field and management of many organizations. Due to the efficiency of the CUSUM and EWMA charts, many other control charts have been developed. Lucas and Crosier [2] incorporated a fast initial response (FIR) feature to the classical CUSUM and compared its performance to that of the classical CUSUM chart. Zhao et al. [3] developed a dual CUSUM scheme which combines two CUSUM chart to detect a small shift in a process. Li and Wang [4] proposed an adaptive CUSUM Q chart also known as the self-starting approach. Shafqat et al. [5] introduced a linear prediction model based on the double EWMA control chart for non-normal situations for monitoring the location parameter. Haq [6] and Haq et al. [7, 8] proposed the use of EWMA control charts to monitor various processes, i.e. the mean and variance using ranked set sampling and the mean under the effect of measurement errors using ranked set sampling. Sanusi et al. [9] introduced a different approach for the EWMA-based chart when supplementary information about the main variable is not constant. Some different applications of attributes and variables control charts can be found in [10, 11].

The classical Shewhart control charts cannot be used efficiently when data are collected from uncertain and random situations, or when there are inconsistencies in the collected data. Khademi and Amirzadeh [12] reported that “fuzzy data exist ubiquitously in the modern manufacturing processes”; hence, in certain monitoring environments, ‘fuzzy model’ control charts are preferred when parameters or observations are uncertain, see for instance [13]. Senturk and Erginel [14] introduced the dispersion control chart by using the fuzzy logic, and Faraz et al. [15] proposed a control chart combining uncertainty and randomness assumptions. Smarandache [16] provided an overview of the fuzzy logic and reported that the neutrosophic logic deals efficiently with indeterminacy. In [17, 18], the neutrosophic logic is used to develop the idea of the neutrosophic statistics. Neutrosophic statistics is an extension of the classical statistics that is more efficient in analyzing data from ambiguous environment. For publications on neutrosophic statistics applied in various fields of knowledge, the readers are referred to [19–21]. For non-normal processes, control charts with a fuzzy approach are discussed by [22]. Pereira et al. [23] designed a fuzzy chart for monitoring the blood components under the violation of the normality assumption. The most recent research works on control charts that use neutrosophic statistics concepts, can be found in [24–28].

At the best of the authors’ knowledge, there is no work on the CUSUM control chart under neutrosophic statistics. In this paper, the CUSUM $\bar{X}$ control chart using the neutrosophic statistical method is proposed. Following the reports in [16, 17], it is presumed that the proposed neutrosophic CUSUM (NCUSUM) $\bar{X}$ chart will provide very interesting properties (i.e. will be more efficient in detecting a small shift in the process and more informative, flexible, and effective in uncertain conditions).

The rest of this paper is structured as follows: The second section gives a brief description of the classical CUSUM $\bar{X}$ chart and an introduction to the NCUSUM $\bar{X}$ control chart. In the third section, the performance of the proposed NCUSUM $\bar{X}$ chart is extensively investigated in terms of the neutrosophic run length properties. Moreover, the performance of the proposed chart is compared with the classical CUSUM $\bar{X}$ chart. Fourth and fifth sections provide illustrative applications of the new chart using simulated and real-life datasets, respectively. The conclusion and recommendations are given in the last section.

### The CUSUM control chart

#### The classical CUSUM chart

The CUSUM $\bar{X}$ chart for monitoring small and moderate shifts in the mean was first introduced by [29]. The two-sided CUSUM chart is used to monitor upwards and downward shifts in the process parameters using the charting statistics $C_{i}^{+}$ and $C_{i}^{-}$, respectively, where *i* represents the sample number (or sampling time). Assume that *X* is a sequence of independent normally distributed observations given that the in-control mean *μ*_0_ and variance $\sigma_{0}^{2}$; thus, the charting statistics of the two-sided CUSUM $\bar{X}$ chart (i.e. $C_{i}^{+}$ and $C_{i}^{-}$, with $C_{0}^{+}=C_{0}^{-}=0$) are plotted versus a single control limit denoted by *H*. These charting statistics are mathematically defined by

$$\left\{ \begin{array}{c} C_{i}^{+}=\max\left[0,{\bar{X}}_{i}-(\mu_{0}+K)+C_{i-1}^{+}\right]\\ C_{i}^{-}=\max\left[0,(\mu_{0}-K)-{\bar{X}}_{i}+C_{i-1}^{-}\right] \end{array}\right.,$$ (1)

where $\bar{X}$ is the sample mean, *μ*_0_ is the target mean value of *X* and *K* is the reference value. The CUSUM chart depends on two singleton parameters, i.e. *K* and *H*, which need to be selected carefully because the sensitivity of the CUSUM chart depend on them, see pages 404–405 of Montgomery [1]. These parameters are chosen such that:

$$K=k\sqrt{Var(\bar{X})}\;\mathrm{and}\;H=h\sqrt{Var(\bar{X})},$$ (2)

where $Var(\bar{X})=\sigma_{X}^{2}/n,\;\sigma_{X}^{2}$ is the variance of *X*, *k* is selected to be half of the shift (i.e. k=δ2, with *δ* is the shift in standard deviation units) and *h* is usually selected to be approximately five times the standard deviation units. Note that the process has moved upwards if $C_{i}^{+}>H$ for any value of *i*; however, if $C_{i}^{-}>H$, the process has moved downwards.

In the next sub-section, the proposed NCUSUM $\bar{X}$ chart is introduced using the framework of the classical CUSUM $\bar{X}$ chart.

#### The proposed neutrosophic CUSUM chart

In the uncertain environments having inconsistency data, the quality characteristic of interest (denoted by *X*_*Ni*_) is not a deterministic value; hence, it falls within some interval, say [*X*_*Li*_, *X*_*Ui*_], where the subscripts *L* and *U* refers to the lower and upper bound of those particular values; for instance, *X*_*Li*_ is a lower bound of *X*_*Ni*_ and *X*_*Ui*_ is the upper bound of *X*_*Ni*_ (i.e. *X*_*Li*_≤*X*_*Ni*_≤*X*_*Ui*_). The latter lower and upper notation holds for other expressions defined in this subsection. That is, the neutrosophic quality characteristic of interest *X*_*Ni*_; *X*_*Ni*_∈[*X*_*Li*_, *X*_*Ui*_], follows neutrosophic normal distribution with neutrosophic mean *μ*_0*N*_; *μ*_0*N*_∈[*μ*_*L*_, *μ*_*U*_] and neutrosophic variance $\sigma_{0N}^{2};\;\sigma_{0N}^{2}\in [\sigma_{L}^{2},\sigma_{U}^{2}]$, with *μ*_*L*_≤*μ*_*U*_ and $\sigma_{L}^{2}\leq \sigma_{U}^{2}$. Hence, the sample mean of the variable *X*_*Ni*_ is given by ${\bar{X}}_{Ni}\in [{\bar{X}}_{Li},{\bar{X}}_{Ui}]$, Var(X¯Ni)=σNi2nN;nN∈[nL,nU] with *n*_*L*_≤*n*_*U*_, *σ*_*Ni*_∈[*σ*_*Li*_, *σ*_*Ui*_] is the observed neutrosophic standard deviation of *X*_*iN*_, with *σ*_*Li*_≤*σ*_*Ui*_. Thus, the plotting statistics of the proposed NCUSUM $\bar{X}$ chart under uncertain environments is given by

$$\left\{ \begin{array}{c} M_{Ni}^{+}=max[0,{\bar{X}}_{Ni}-(\mu_{0N}+K_{N})+M_{N(i-1)}^{+}]\\ M_{Ni}^{-}=\max\left[0,(\mu_{0N}-K_{N})-{\bar{X}}_{Ni}+M_{N(i-1)}^{-}\right] \end{array}\right.$$ (3)

where $M_{Ni}^{+}\in [M_{Li}^{+},M_{Ui}^{+}]$, $M_{Ni}^{-}\in [M_{Li}^{-},M_{Ui}^{-}]$, *K*_*N*_∈[*K*_*L*_, *K*_*U*_], *H*_*N*_∈[*H*_*L*_, *H*_*U*_] and *μ*_0*N*_ is the target mean value. In addition, *μ*_1*N*_ = *μ*_0*N*_+*δσ*_0*N*_, where *μ*_0*N*_∈[*μ*_0*L*_, *μ*_0*U*_] and *μ*_1*N*_∈[*μ*_1*L*_, *μ*_1*U*_] represents the in-control and out-of-control neutrosophic mean, respectively; with *δ* denoting the shift in the mean. Moreover,

$$K_{N}=k_{N}\sqrt{Var({\bar{X}}_{N})}\;\mathrm{and}\;H_{N}=h_{N}\sqrt{Var({\bar{X}}_{N})};\;k_{N}\in [k_{L},k_{U}];\;h_{N}\in [h_{L},h_{U}],$$ (4)

where *k*_*N*_ and *h*_*N*_ are the design parameters of the proposed NCUSUM $\bar{X}$ chart. The initial values for the plotting statistics which are given in Eq (3) are equal to zero, i.e. $M_{N0}^{+}=M_{N0}^{-}=0$. The plotting statistics of the proposed chart are plotted versus the control limit *H*_*N*_. The evaluation rule for the proposed chart is given as: for any value of *i*, if the value of $M_{Ni}^{+}>H_{N}$ then the process of the mean is declared to have shifted upward; otherwise, if the value of $M_{Ni}^{-}>H_{N}$ then the process mean is declared to have moved downward. The values of *k*_*N*_ and *h*_*N*_ need to be selected with caution because the run length (*RL*) properties of the chart depend on them. Note that the *RL* is the number of charting statistics that are required before the first out-of-control signal is issued by a control chart. The manner in which the design parameters *k*_*N*_ and *h*_*N*_ are obtained is discussed in the next section.

### Run length performance

#### Performance of the NCUSUM $\bar{X}$ chart

For some selected values of *k*_*N*_ and *n*_*N*_, for example *k*_*N*_∈{[0.20,0.25], [0.4,0.5]}, and *n*_*N*_∈{[3,5],[10,20]}, the corresponding values of *h*_*N*_ are found by running 10^5^ simulations in R software for some given fixed nominal neutrosophic average *RL* (denoted by *NARL*_0_) values of 300, 370, 400, 500. Tables 1–3 display the in-control and out-of-control neutrosophic average and standard deviation of the *RL* (denoted as *NARL* and *NSDRL*) profiles of the proposed chart using different parameters, where *NARL*∈[*NARL*_*L*_, *NARL*_*U*_] and *NSDRL*∈[*NSDRL*_*L*_, *NSDRL*_*U*_], with *NARL*_*L*_≤*NARL*_*U*_ and *NSDRL*_*L*_≤*NSDRL*_*U*_. In other words, Tables 1–3 give the ideal combinations of *h*_*N*_ and *k*_*N*_ when the nominal *NARL*_0_ values are 300, 370, 400 and 500 for different values of *n*_*N*_.

**Table 1:** The *NARL* and *NSDRL* values of the proposed NCUSUM $\bar{X}$ chart when *n*_*N*_∈[3, 5], *k*_*N*_∈[0.20, 0.25] for different nominal *NARL*.

*NARL*	300		370		400		500	
*h*_*N*_	[7.602,8.662]		[8.099,9.299]		[8.319,9.499]		[9.039,10.299]	
*δ*	*NARL*s	*NSDRL*s	*NARL*s	*NSDRL*s	*NARL*s	*NSDRL*s	*NARL*s	*NSDRL*s
0	[301.07,301.18]	[271.92,283.10]	[370.28,371.70]	[330.58,342.12]	[400.39,401.79]	[360.15,371.83]	[500.44,501.54]	[441.99,447.12]
0.25	[22.74,32.70]	[12.50,19.00]	[24.94,35.23]	[13.38,20.73]	[25.64,36.61]	[13.68,20.68]	[27.04,40.06]	[14.01,22.71]
0.5	[9.11,13.77]	[3.38,5.35]	[9.77,14.57]	[3.32,5.38]	[10.20,15.04]	[3.49,5.47]	[11.15,15.74]	[3.61,5.58]
0.75	[5.00,8.59]	[1.61,2.56]	[6.36,9.33]	[1.75,2.80]	[6.54,9.52]	[1.77,2.66]	[6.96,10.14]	[1.76,2.78]
1	[4.24,6.27]	[1.06,1.60]	[4.68,6.67]	[1.09,1.69]	[4.82,6.87]	[1.06,1.71]	[5.20,7.36]	[1.15,1.71]
1.5	[2.99,4.21]	[0.58,0.87]	[3.17,4.51]	[0.57,0.87]	[3.24,4.51]	[0.61,0.89]	[3.44,4.86]	[0.61,0.91]
2	[2.25,3.22]	[0.44,0.57]	[2.44,3.38]	[0.50,0.60]	[2.46,3.42]	[0.51,0.59]	[2.67,3.70]	[0.51,0.60]
2.5	[1.99,2.60]	[0.33,0.51]	[2.03,2.81]	[0.29,0.56]	[2.03,2.84]	[0.20,0.56]	[2.14,3.03]	[0.34,0.49]
3	[1.87,2.18]	[0.15,0.38]	[1.96,2.29]	[0.19,0.45]	[1.96,2.38]	[0.17,0.46]	[1.99,2.60]	[0.29,0.39]
4	[1.15,1.97]	[0.16,0.35]	[1.26,1.99]	[0.08,0.43]	[1.36,2.00]	[0.09,0.44]	[1.62,2.01]	[0.18,0.30]
5	[1.00,1.57]	[0.00,0.29]	[1.00,1.80]	[0.03,0.39]	[1.00,1.85]	[0.05,0.40]	[1.02,1.96]	[0.15,0.25]
6	[1.00,1.07]	[0.00,0.16]	[1.00,1.20]	[0.00,0.20]	[1.00,1.22]	[0.01,0.22]	[1.00,1.55]	[0.00,0.23]
7	[1.00,1.00]	[0.00,0.03]	[1.00,1.00]	[0.00,0.07]	[1.00,1.00]	[0.00,0.09]	[1.00,1.03]	[0.00,0.12]

**Table 2:** The *NARL* and *NSDRL* values of the proposed NCUSUM $\bar{X}$ chart when *n*_*N*_∈[3, 5], *k*_*N*_∈[0.4, 0.5] for different nominal NARL.

*NARL*_0_	300		370		400		500	
*h*_*N*_	[4.585,5.421]		[4.787,5.751]		[4.887,5.894]		[5.179,6.314]	
*δ*	*NARL*s	*NSDRL*s	*NARL*s	*NSDRL*s	*NARL*s	*NSDRL*s	*NARL*s	*NSDRL*s
0	[301.20,301.83]	[292.12,299.37]	[370.88,371.32]	[354.69,355.21]	[400.82,401.85]	[370.48,374.30]	[501.32,501.62]	[450.81,452.90]
0.25	[25.76,35.74]	[20.24,27.67]	[28.53,41.29]	[22.35,32.16]	[28.51,43.22]	[21.76,33.36]	[31.94,48.29]	[25.25,37.55]
0.5	[8.07,12.05]	[4.07,6.65]	[8.27,12.84]	[4.07,6.84]	[8.38,13.11]	[2.98,6.43]	[9.13,13.70]	[4.53,6.49]
0.75	[4.61,6.67]	[1.74,2.60]	[4.70,7.09]	[1.69,2.72]	[4.87,7.20]	[1.77,2.68]	[5.08,7.65]	[1.78,2.91]
1	[3.27,4.79]	[0.99,1.64]	[3.35,5.06]	[0.99,1.64]	[3.41,5.15]	[1.04,1.69]	[3.55,5.46]	[1.04,1.63]
1.5	[2.17,3.04]	[0.47,0.81]	[2.24,3.15]	[0.52,0.81]	[2.25,3.28]	[0.52,0.79]	[2.35,3.51]	[0.55,0.86]
2	[1.74,2.29]	[0.45,0.51]	[1.81,2.43]	[0.40,0.53]	[1.85,2.45]	[0.40,0.55]	[1.91,2.61]	[0.36,0.58]
2.5	[1.32,1.98]	[0.32,0.46]	[1.36,2.03]	[0.32,0.48]	[1.43,2.05]	[0.30,0.32]	[1.49,2.12]	[0.30,0.37]
3	[1.04,1.74]	[0.19,0.43]	[1.08,1.82]	[0.27,0.38]	[1.08,1.86]	[0.27,0.34]	[1.15,1.93]	[0.25,0.30]
4	[1.00,1.12]	[0.00,0.33]	[1.00,1.21]	[0.00,0.20]	[1.02,1.26]	[0.00,0.30]	[1.00,1.42]	[0.09,0.19]
5	[1.00,1.00]	[0.00,0.00]	[1.00,1.00]	[0.00,0.07]	[1.00,1.01]	[0.00,0.09]	[1.00,1.02]	[0.00,0.06]
6	[1.00,1.00]	[0.00,0.00]	[1.00,1.00]	[0.00,0.00]	[1.00,1.00]	[0.00,0.00]	[1.00,1.00]	[0.00,0.00]
7	[1.00,1.00]	[0.00,0.00]	[1.00,1.00]	[0.00,0.00]	[1.00,1.00]	[0.00,0.00]	[1.00,1.00]	[0.00,0.00]

**Table 3:** The *NARL* and *NSDRL* values of the proposed NCUSUM $\bar{X}$ chart when *n*_*N*_∈[10, 20], *k*_*N*_∈[0.4, 0.5] for different nominal *NARL*.

*NARL*_0_	300		370		400		500	
*h*_*N*_	[5.478,7.389]		[4.804,5.730]		[4.917,5.859]		[5.224,6.172]	
*δ*	*NARL*s	*NSDRL*s	*NARL*s	*NSDRL*s	*NARL*s	*NSDRL*s	*NARL*s	*NSDRL*s
0	[301.10,301.60]	[282.31,301.61]	[370.27,370.54]	[353.60,359.17]	[401.29,401.47]	[374.27,385.41]	[500.08,501.92]	[453.29,460.42]
0.25	[12.32,13.55]	[3.84,6.87]	[8.43,14.49]	[4.28,8.06]	[8.50,14.96]	[4.02,7.65]	[9.09,15.13]	[4.12,7.73]
0.5	[4.89,5.46]	[0.97,1.80]	[3.45,5.51]	[1.06,1.94]	[3.47,5.65]	[1.01,1.88]	[3.67,6.11]	[1.08,2.07]
0.75	[3.16,3.29]	[0.49,0.94]	[2.23,3.50]	[0.51,0.90]	[2.27,3.62]	[0.49,0.96]	[2.39,3.74]	[0.56,0.99]
1	[2.32,2.86]	[0.45,0.61]	[1.81,2.67]	[0.41,0.61]	[1.84,2.68]	[0.40,0.64]	[1.92,2.79]	[0.33,0.65]
1.5	[1.63,1.87]	[0.23,0.34]	[1.08,1.95]	[0.27,0.37]	[1.09,1.94]	[0.28,0.58]	[1.17,2.00]	[0.25,0.37]
2	[1.02,1.14]	[0.00,0.27]	[1.00,1.41]	[0.03,0.25]	[1.00,1.49]	[0.00,0.50]	[1.00,1.58]	[0.00,0.49]
2.5	[1.00,1.00]	[0.00,0.14]	[1.00,1.06]	[0.00,0.20]	[1.00,1.04]	[0.00,0.20]	[1.00,1.09]	[0.00,0.28]
3	[1.00,1.00]	[0.00,0.00]	[1.00,1.00]	[0.00,0.03]	[1.00,1.00]	[0.00,0.04]	[1.00,1.00]	[0.00,0.03]
4	[1.00,1.00]	[0.00,0.00]	[1.00,1.00]	[0.00,0.00]	[1.00,1.00]	[0.00,0.00]	[1.00,1.00]	[0.00,0.00]
5	[1.00,1.00]	[0.00,0.00]	[1.00,1.00]	[0.00,0.00]	[1.00,1.00]	[0.00,0.00]	[1.00,1.00]	[0.00,0.00]
6	[1.00,1.00]	[0.00,0.00]	[1.00,1.00]	[0.00,0.00]	[1.00,1.00]	[0.00,0.00]	[1.00,1.00]	[0.00,0.00]
7	[1.00,1.00]	[0.00,0.00]	[1.00,1.00]	[0.00,0.00]	[1.00,1.00]	[0.00,0.00]	[1.00,1.00]	[0.00,0.00]

The following algorithm was applied to determine the values of *k*_*N*_ and *n*_*N*_.

**Step-1:** specify the values of *NARL*_0_ and *n*_*N*_.
**Step-2:** Determine the values of *k*_*N*_ and *n*_*N*_ where *NARL*≥*NARL*_0_. Chose the values of *k*_*N*_ and *n*_*N*_ where *NARL* is very close or exact to *NARL*_0_.
**Step-3:** Determine the values of NARLs and NSDRLs for various values of *δ*.

The performance of the proposed NCUSUM $\bar{X}$ control chart is evaluated in terms of different *RL* criteria, i.e. *NARL*s, *NSDRL* and the percentiles of the run length (*PRL*), i.e. the 25^th^, 50^th^ and 75^th^ percentiles which are denoted by P25 (i.e. *P*25∈[*P*25_*L*_, *P*25_*U*_]), P50 (i.e. *P*50∈[*P*50_*L*_, *P*50_*U*_]) and P75 (i.e. *P*75∈[*P*75_*L*_, *P*75_*U*_]). The following analysis would be noticed through Tables 1–6:

1. Tables 1–5 show that there are inverse relationships between the values of out-of-control *NARL* (denoted as *NARL*_1_) and the values of *δ*. That is, the smallest value of *NARL*_1_ is found at the largest values of *δ*, i.e. [1.00,1.00]. In other words, as *δ* increase, the values of *NARL*_1_ decrease until they approach a value of one, i.e. [1.00,1.00]. The latter implies that, for large values of *δ*, the control chart will give an out-of-control signal on the next sampling point. Similarly, the *NSDRL*s tend to zero for largest values of *δ*, i.e. [0.00,0.00], which means that the variability is reduced when *δ* values are large.
2. For a specified neutrosophic sample size, *n*_*N*_, there is an increasing trend in *NARL*_1_ values as the neutrosophic value of *k*_*N*_ increase at small shifts of *δ*; however, there is an decreasing trend in *NARL*_1_ values for large shifts of *δ* (see Tables 1 and 2). For instance, when *k*_*N*_∈[0.20,0.25], *n*_*N*_∈[3,5] and *δ* = 0.25 for a *NARL*_0_ = 370, the *NARL*_1_∈[24.94,35.23]; however, when *k*_*N*_∈[0.40,0.50], the *NARL*_1_∈[28.53,41.29] using the same parameters. Note though, at *δ* = 3 for the same *NARL*_0_ value, the *NARL*_1_ is equal to [1.96,2.29] and [1.08,1.82] for *k*_*N*_ equal to [0.20,0.25] and [0.40,0.50], respectively. Moreover, when design parameters are kept fixed, as *δ* increase, there is a decrease in the values of *NARL*_1_.
3. From Tables 2 and 3, it can be noticed that the *NARL*_1_ decreases rapidly when *n*_*N*_ increase, with the restriction for the value of [*k*_*L*_, *k*_*U*_] being constant. For instance, when *δ* = 0.25 and [*k*_*L*_, *k*_*U*_] = [0.40,0.50] for a nominal *NARL*_0_ = 370, the *NARL*_1_ is equal to [28.53,41.29] and [8.43,14.49] when *n*_*N*_ is equal to [3,5] and [10,20], respectively. This indicates improving detection ability.
4. The in-control neutrosophic *RL* distribution of the proposed chart is positively skewed as the in-control *NARL* are greater than the in-control *P*50. For instance, for the design parameters *k*_*N*_∈[0.20,0.25] and *n*_*N*_∈[3,5] corresponding to *δ* = 0, the in-control values of the *P*50∈[273,277] are smaller than *NARL*_0_ = 370 (see for instance Tables 1–6). The latter implies that the distribution of the NCUSUM $\bar{X}$ chart is positively skewed.
5. Regardless of the *NARL*_0_ value, the sensitivity of the NCUSUM $\bar{X}$ chart increase rapidly as *δ* increases in terms of the P25, P50 and P75 values, see Tables 4–6.

**Table 4:** The neutrosophic percentile points of the proposed NCUSUM $\bar{X}$ chart when *n*_*N*_∈[3, 5], *k*_*N*_∈[0.20, 0.25] for different nominal *NARL*.

*NARL*_0_	300		370		400		500	
*δ*	P25	P50	P25	P75	P50	P75	P25	P50
0	[98,104]	[220,230]	[111,123]	[399,420]	[273,277]	[502,518]	[119,132]	[293,294]
0.25	[14,19]	[21,28]	[15,21]	[28,41]	[22,30]	[31,44]	[16,22]	[23,31]
0.5	[7,10]	[9,13]	[7,11]	[11,17]	[9,14]	[12,18]	[8,11]	[10,14]
0.75	[5,7]	[6,8]	[5,7]	[7,10]	[6,9]	[7,11]	[5,8]	[6,9]
1	[4,5]	[4,6]	[4,5]	[5,7]	[5,6]	[5,8]	[4,6]	[5,7]
1.5	[3,4]	[3,4]	[3,4]	[3,5]	[3,4]	[3,5]	[3,4]	[3,4]
2	[2,3]	[2,3]	[2,3]	[3,4]	[2,3]	[3,4]	[2,3]	[2,3]
2.5	[2,2]	[2,3]	[2,3]	[2,3]	[2,3]	[2,3]	[2,3]	[2,3]
3	[2,2]	[2,2]	[2,2]	[2,2]	[2,2]	[2,3]	[2,2]	[2,2]
4	[1,2]	[1,2]	[1,2]	[1,2]	[1,2]	[2,2]	[1,2]	[1,2]
5	[1,1]	[1,2]	[1,2]	[1,2]	[1,2]	[1,2]	[1,2]	[1,2]
6	[1,1]	[1,1]	[1,1]	[1,1]	[1,1]	[1,1]	[1,1]	[1,1]
7	[1,1]	[1,1]	[1,1]	[1,1]	[1,1]	[1,1]	[1,1]	[1,1]

**Table 5:** The neutrosophic percentile points of the proposed NCUSUM $\bar{X}$ chart when *n*_*N*_∈[3, 5], *k*_*N*_∈[0.4, 0.5] for different nominal *NARL*.

*NARL*_0_	300		370		400		500	
*δ*	P25	P50	P25	P75	P50	P75	P25	P50
0	[87,91]	[201,202]	[109,109]	[405,428]	[264,268]	[504,528]	[120,120]	[285,293]
0.25	[12,17]	[19,27]	[12,18]	[34,47]	[22,33]	[37,54]	[13,19]	[22,33]
0.5	[5,7]	[7,10]	[5,8]	[10,15]	[7,11]	[10,16]	[6,8]	[7,12]
0.75	[3,5]	[4,6]	[3,5]	[6,8]	[4,7]	[6,8]	[4,5]	[5,7]
1	[3,4]	[3,5]	[3,4]	[4,6]	[3,5]	[4,6]	[3,4]	[3,5]
1.5	[2,3]	[2,3]	[2,3]	[2,3]	[2,3]	[3,4]	[2,3]	[2,3]
2	[1,2]	[2,2]	[2,2]	[2,3]	[2,2]	[2,3]	[2,2]	[2,2]
2.5	[1,2]	[1,2]	[1,2]	[2,2]	[1,2]	[2,2]	[1,2]	[1,2]
3	[1,1]	[1,2]	[1,2]	[1,2]	[1,2]	[1,2]	[1,2]	[1,2]
4	[1,1]	[1,1]	[1,1]	[1,1]	[1,1]	[1,1]	[1,1]	[1,1]
5	[1,1]	[1,1]	[1,1]	[1,1]	[1,1]	[1,1]	[1,1]	[1,1]
6	[1,1]	[1,1]	[1,1]	[1,1]	[1,1]	[1,1]	[1,1]	[1,1]
7	[1,1]	[1,1]	[1,1]	[1,1]	[1,1]	[1,1]	[1,1]	[1,1]

**Table 6:** The neutrosophic percentile points of the proposed NCUSUM $\bar{X}$ chart when *n*_*N*_∈[10, 20], *k*_*N*_∈[0.4, 0.5] for different nominal *NARL*.

*NARL*_0_	300		370		400		500	
*δ*	P25	P50	P25	P75	P50	P75	P25	P50
0.00	[89,90]	[201,205]	[111,114]	[408,408]	[250,251]	[511,547]	[120,121]	[275,276]
0.25	[5,8]	[7,12]	[5,9]	[10,17]	[8,12]	[10,18]	[6,9]	[8,13]
0.5	[3,4]	[3,5]	[3,4]	[4,6]	[3,5]	[4,6]	[3,4]	[3,5]
0.75	[2,3]	[2,3]	[2,3]	[2,4]	[2,3]	[3,4]	[2,3]	[2,3]
1	[1,2]	[2,2]	[2,2]	[2,3]	[2,3]	[2,3]	[2,2]	[2,3]
1.5	[1,2]	[1,2]	[1,2]	[1,2]	[1,2]	[1,2]	[1,2]	[1,2]
2	[1,1]	[1,1]	[1,1]	[1,2]	[1,1]	[1,2]	[1,1]	[1,1]
2.5	[1,1]	[1,1]	[1,1]	[1,1]	[1,1]	[1,1]	[1,1]	[1,1]
3	[1,1]	[1,1]	[1,1]	[1,1]	[1,1]	[1,1]	[1,1]	[1,1]
4	[1,1]	[1,1]	[1,1]	[1,1]	[1,1]	[1,1]	[1,1]	[1,1]
5	[1,1]	[1,1]	[1,1]	[1,1]	[1,1]	[1,1]	[1,1]	[1,1]
6	[1,1]	[1,1]	[1,1]	[1,1]	[1,1]	[1,1]	[1,1]	[1,1]
7	[1,1]	[1,1]	[1,1]	[1,1]	[1,1]	[1,1]	[1,1]	[1,1]

#### Comparison of the proposed NCUSUM $\bar{X}$ and classical CUSUM $\bar{X}$ charts

The persistent need for more awareness of the proposed NCUSUM $\bar{X}$ chart, comprehensive comparison has been made with classical CUSUM $\bar{X}$ chart. To get valid deductions, the in-control *ARL*s of both charts are set at nominal levels of 370 and 500, and the performance is compared in terms of the out-of-control *ARL*s and *SDRL*s. The strategy of working in this section is parallel to [27], that is, a control chart under neutrosophic design is said to be capable if it has smaller *NARL* values compared to its counterpart. Thus, the efficiency of the proposed chart in terms of the *NARL* values is compared to the *ARL* values of the classical CUSUM $\bar{X}$ chart. For instance, the classical CUSUM $\bar{X}$ chart with parameters *k* = 0.5 and *h* = 4.822 identifies a shift of size equal to 0.25 after 125.27 charting statistics for an *ARL*_0_ of 370; however, the proposed NCUSUM $\bar{X}$ chart with parameters *k*_*N*_∈[0.4,0.5] and *h*_*N*_∈[4.887,5.894] identifies a shift of size equal to 0.25 between the interval [28.53,41.29] samples for an *NARL*_0_ of 370. In general, the comparison in Table 7 indicates that for small, moderate and large shifts in the process mean, the *NARL* and *NSDRL* are smaller in magnitude as compared to the *ARL* and *SDRL* of the classical chart, at each corresponding shift value; and thus, the proposed NCUSUM $\bar{X}$ chart performs better than the classical CUSUM $\bar{X}$ chart. In summary, the neutrosophic and classical *ARL*s and *SDRL*s of the NCUSUM and CUSUM $\bar{X}$ charts indicate that the NCUSUM $\bar{X}$ chart has the higher shift detection ability as compared to the classical CUSUM $\bar{X}$ chart.

**Table 7:** Comparison of the NCUSUM and the classical CUSUM (CCUSUM) $\bar{X}$ charts when *k* = 0.5, *k*_*N*_∈[0.4, 0.5].

	CCUSUM chart: *ARL*_0_ = 370		NCUSUM chart: *NARL*_0_ = 370		CCUSUM chart: *ARL*_0_ = 500		NCUSUM chart: *NARL*_0_ = 500	
	h = 4.822		*n*_*N*_∈[3, 5], *h*_*N*_∈[4.887, 5.894]		h = 5.296		*n*_*N*_∈[3, 5], *h*_*N*_∈[5.224, 6.172]	
*δ*	*ARL*s	*SDRL*s	*NARL*s	*NSDRL*s	*ARL*s	*SDRL*s	*NARL*s	*NSDRL*s
0	370.11	354.62	[370.88,371.32]	[354.69,355.21]	500.86	458.86	[501.32,501.62]	[501.32,501.62]
0.25	125.27	122.52	[28.53,41.29]	[22.35,32.16]	162.99	150.86	[31.94,48.29]	[48.29,31.94]
0.5	35.37	28.00	[8.27,12.84]	[4.07,6.84]	43.01	33.77	[9.13,13.70]	[13.70,9.13]
0.75	16.39	10.48	[4.70,7.09]	[1.69,2.72]	18.15	11.89	[5.08,7.65]	[7.65,5.08]
1	9.98	5.25	[3.35,5.06]	[0.99,1.64]	11.46	6.37	[3.55,5.46]	[5.46,3.55]
1.5	5.60	2.18	[2.24,3.15]	[0.52,0.81]	5.96	2.19	[2.35,3.51]	[3.51,2.35]
2	3.85	1.23	[1.81,2.43]	[0.40,0.53]	4.19	1.27	[1.91,2.61]	[2.61,1.91]
2.5	3.00	0.85	[1.36,2.03]	[0.32,0.48]	3.26	0.89	[1.49,2.12]	[2.12,1.49]
3	2.48	0.63	[1.08,1.82]	[0.27,0.38]	2.72	0.71	[1.15,1.93]	[1.93,1.15]
4	1.95	0.37	[1.00,1.21]	[0.00,0.20]	2.07	0.38	[1.00,1.42]	[1.42,1.00]
5	1.62	0.48	[1.00,1.00]	[0.00,0.07]	1.80	0.40	[1.00,1.02]	[1.02,1.00]
6	1.24	0.43	[1.00,1.00]	[0.00,0.00]	1.40	0.49	[1.00,1.00]	[1.00,1.00]
7	1.04	0.19	[1.00,1.00]	[0.00,0.00]	1.11	0.31	[1.00,1.00]	[1.00,1.00]

### Application of the NCUSUM $\bar{X}$ chart using simulated data

**Fig 1:**
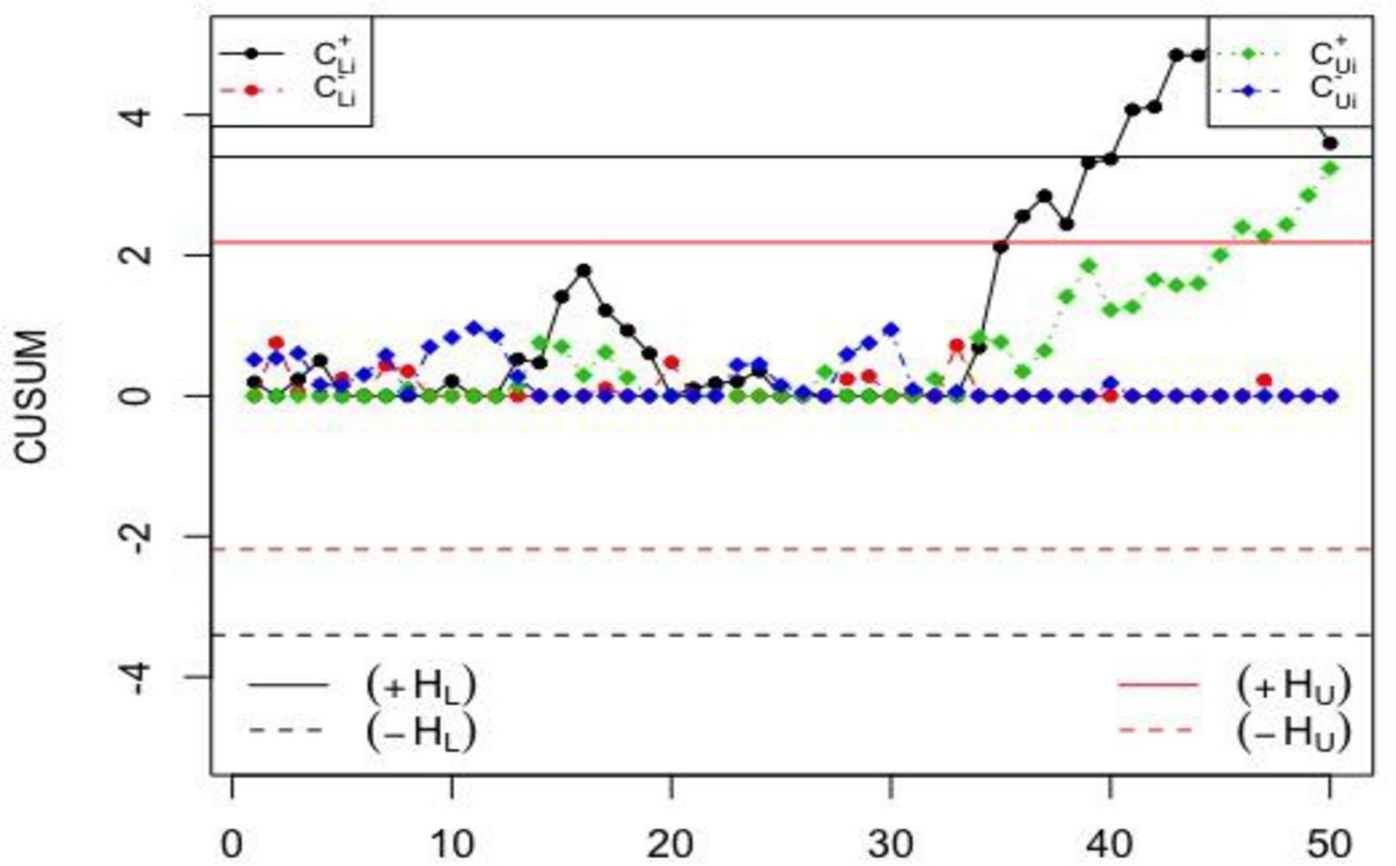
The proposed NCUSUM $\bar{X}$ chart for simulated dataset.

**Fig 2:**
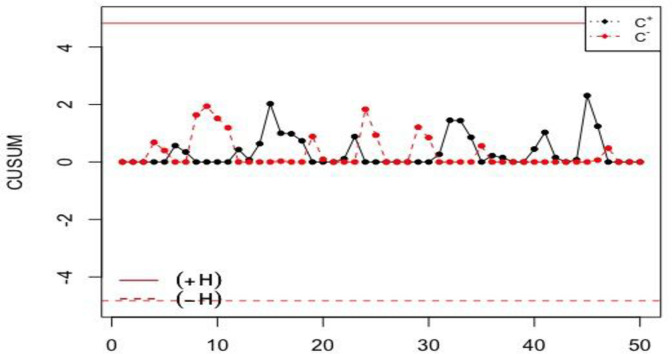
The classical CUSUM $\bar{X}$ chart for simulated dataset.

In this section, the performance of the NCUSUM $\bar{X}$ chart is compared to one of the classical CUSUM $\bar{X}$ chart using simulated data. Fifty observations are generated using the neutrosophic normal distribution. The first twenty observations are generated by assuming that the process is statistically in-control and the next 30 observations are generated by assuming that the process has shifted with *δ* = 0.25. The simulated data along with *n*_*N*_∈[3,5], *k*_*N*_∈[0.4,0.5], *h*_*N*_∈[4.887,5.894], *σ*_0*N*_∈[1.00,1.00], and *NARL*_1_∈[28.53,41.29], so the shift should be detected between the 28^th^ and 41^st^ samples. The results from the simulated data are displayed in Figs 1 and 2 for the proposed NCUSUM chart and the existing classical CUSUM $\bar{X}$ chart, respectively.

From Figs 1 and 2, it can be seen that the proposed NCUSUM $\bar{X}$ chart detects the first shift at around the 30^th^ sample in between the 28^th^ and 41^st^ samples; however, the classical CUSUM $\bar{X}$ chart indicates that all the charting statistics are in-control. Hence, it can be deduced that, the proposed NCUSUM $\bar{X}$ chart has the ability to detect the shifts in the process mean earlier as compared to its classical counterpart. Therefore, the NCUSUM $\bar{X}$ chart is more efficient and performs better than the classical CUSUM $\bar{X}$ chart.

### Real-data application of the proposed CUSUM $\bar{X}$ chart

This section presents case-studies for the NCUSUM $\bar{X}$ chart that were applied to two types of data; the first example was dedicated to the case-study on the Pakistan state oil (PSO), while the second example is the case-study on the weather forecasting. The data were taken from the Stock Exchange and Meteorological Department of Pakistan. However, as mentioned in [28], all the observations and measurements of the current variables are not crisp numbers; they are neutrosophically varied with some indeterminacy in representing intervals instead of singleton numbers.

#### Petroleum industry case-study (PSO)

**Fig 3:**
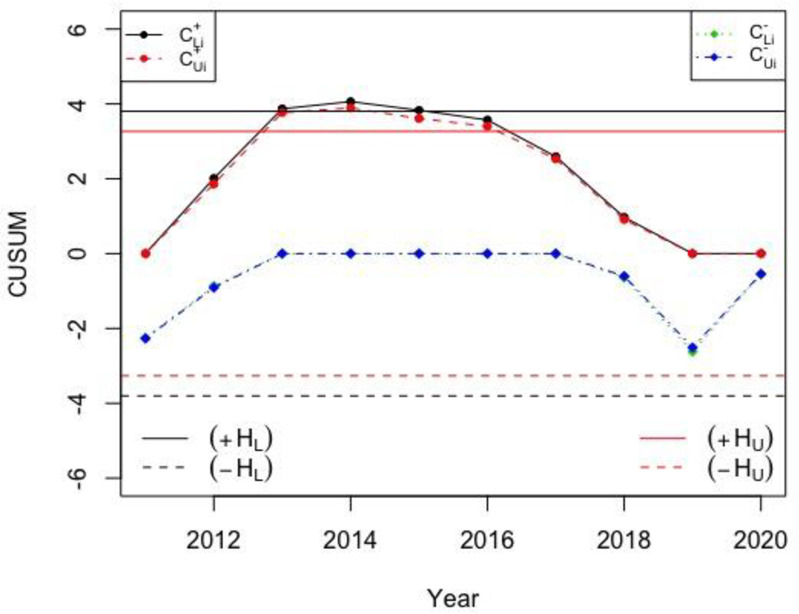
Real-life data from PSO petroleum oil company’s stock price for the proposed NCUSUM $\bar{X}$ chart with *H*_*N*_∈[3.265, 3.803] and *K*_*N*_∈[0.3397, 0.3958].

**Fig 4:**
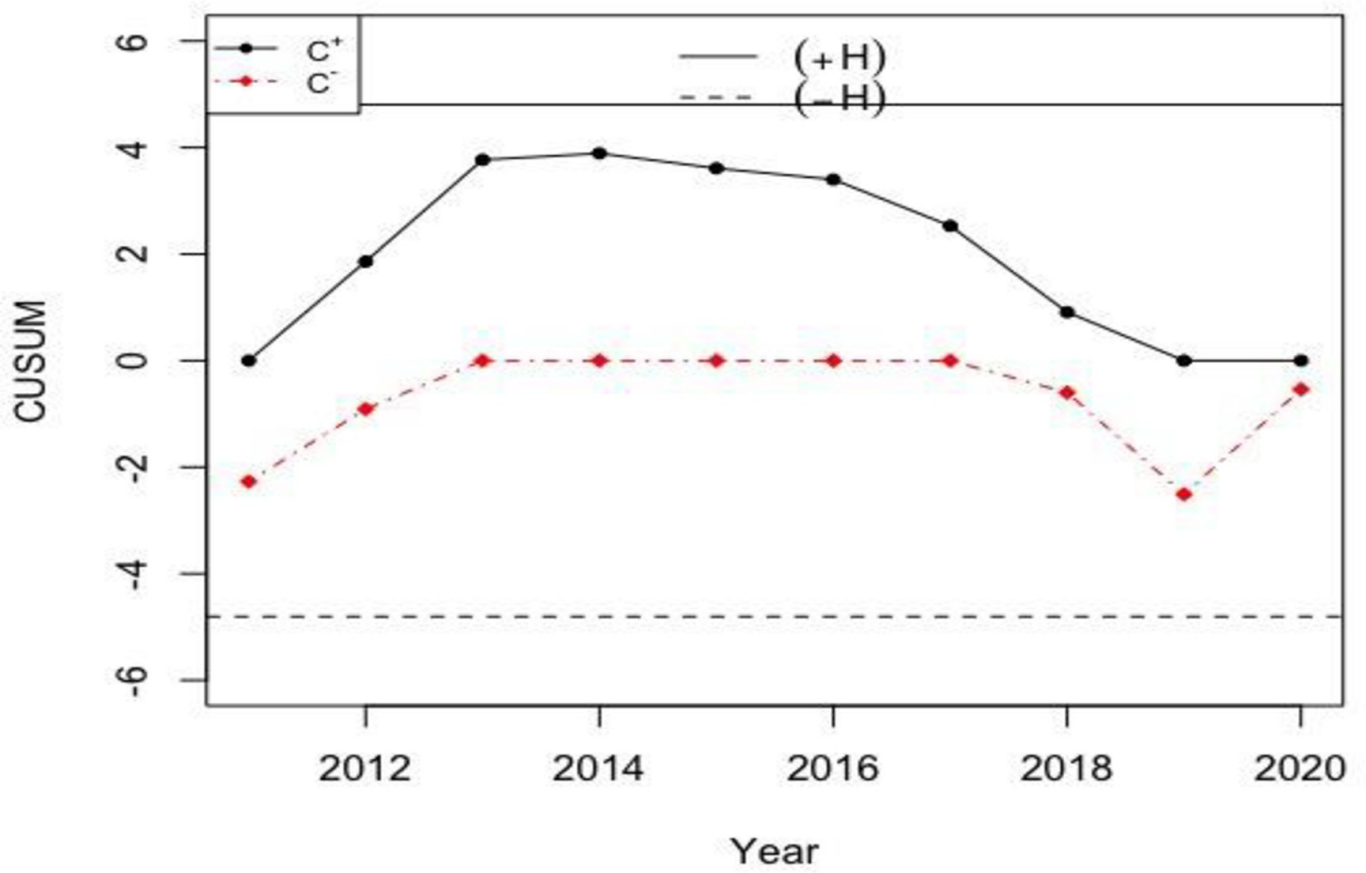
Real-life data from PSO petroleum company’s stock price for the classical CUSUM $\bar{X}$ chart with *h* = 4.804 and *k* = 0.5.

The oil prices are strongly related with the stock prices, which is dependent on the principle of demand and there are some indeterminacy in its measurements. The dataset in Table 8 denotes the stock price in 8 different days of August of the last 10 years for the PSO. For this data, let *n*_*N*_ = 8 and *NARL*_0_ = 370. The reference value $K_{N}={0.5\sigma }_{0N}/\sqrt{n_{N}}$ (i.e. *k* = 0.5), and decision interval $H_{N}={h\sigma }_{0N}/\sqrt{n_{N}}$, where *σ*_0*N*_∈[1.9221,2.239], *n*_*N*_ = 8 and *h* = 4.804 are determined by using the procedure used to derive the values in Tables 1–3. Thus, the calculated decision interval and reference value given in Eq (4) is *H*_*N*_∈[3.265,3.803] and *K*_*N*_∈[0.3397.0.3958]. The calculated charting statistics of the NCUSUM chart (i.e. $M_{Ni}^{+}$ and $M_{Ni}^{-}$) according to Eq (3) are provided in the last two columns of Table 8 and also plotted in Fig 3; however, those of the existing classical CUSUM $\bar{X}$ chart are plotted in Fig 4.

**Table 8:** Real-life example based on the PSO petroleum company’s stock price where *h* = 4.804, *k* = 0.5, *n* = 8, *H*_*N*_∈[3.265, 3.803], *μ*_*N*_∈[226.897, 232.9635] and *σ*_*N*_∈[1.9221, 2.239].

	PSO petroleum oil company’s stock price										
Year	AUGD10	AUGD11	AUGD12	AUGD13	AUGD17	AUGD18	AUGD19	AUGD20	$\left[{\bar{X}}_{iL},{\bar{X}}_{iU}\right]$	$\left[M_{Li}^{+},M_{Ui}^{+}\right]$	$\left[M_{Li}^{-},M_{Ui}^{-}\right]$
2020	[182.00,185.25]	[184.6,190.00]	[184.01,190.52]	[182.9,185.75]	[185.51,197.00]	[191.5,197.00]	[190.51,194.75]	[190.0,194.99]	[186.37,191.90]	[0.00,0.00]	[-0.56,-0.54]
2019	[132.0,138.01]	[132.0,138.01]	[132.0,138.01]	[132.0,138.01]	[127.26,132.00]	[127.26,132.00]	[126.25,134.24]	[132.5,137.79]	[130.16,136.01]	[0.00,0.00]	[-2.62,-2.51]
2018	[279.17,283.24]	[279.17,283.24]	[279.17,283.24]	[288.71,294.62]	[284.57,290.25]	[284.57,290.25]	[284.57,290.25]	[283.33,292.08]	[282.91,288.39]	[0.91,0.97]	[-0.64,-0.60]
2017	[318.06,329.86]	[311.11,320.83]	[311.11,320.83]	[311.11,320.83]	[304.19,311.73]	[301.04,309.38]	[301.04,309.38]	[301.04,309.38]	[307.33,316.52]	[2.53,2.59]	[0.00,0.00]
2016	[286.46,290.90]	[285.08,287.50]	[285.08,288.19]	[285.08,288.19]	[284.03,288.19]	[281.25,284.73]	[279.52,283.82]	[279.52,283.82]	[283.25,286.91]	[3.40,3.57]	[0.00,0.00]
2015	[261.81,267.08]	[263.87,267.63]	[261.81,265.28]	[256.25,264.58]	[250.83,257.64]	[252.86,256.04]	[250.38,256.25]	[244.62,250.69]	[255.31,260.64]	[3.61,3.83]	[0.00,0.00]
2014	[265.69,269.79]	[253.67,267.02]	[247.58,256.24]	[248.73,259.73]	[254.31,263.67]	[258.86,268.48]	[254.25,261.81]	[254.86,263.53]	[254.74,263.77]	[3.89,4.06]	[0.00,0.00]
2013	[228.47,244.73]	[228.47,244.73]	[244.73,251.03]	[245.87,250.63]	[240.35,245.98]	[240.35,245.98]	[237.23,249.46]	[243.23,251.03]	[238.58,247.94]	[3.77,3.87]	[0.00,0.00]
2012	[175.14,177.57]	[175.14,177.57]	[175.14,177.57]	[175.00,180.56]	[174.79,176.39]	[174.79,176.39]	[174.79,176.39]	[174.79,176.39]	[174.94,177.35]	[1.86,2.00]	[-0.91,-0.87]
2011	[145.94,149.97]	[148.61,157.46]	[157.64,163.19]	[157.64,163.19]	[165.01,167.71]	[160.42,165.28]	[153.92,157.23]	[153.92,157.23]	[155.38,160.15]	[0.00,0.00]	[-2.27,-2.25]

From Fig 3, it can be noted that the PSO petroleum stock price are out-of-control in 2013 to 2015 (i.e. higher prices); while, in 2011 and 2012 the petroleum oil prices are in-control (within reasonable range). However, after 2015, the prices are decreasing and are in-control. From Fig 4, it can be seen that in all the monitored years, the PSO petroleum prices are in-control. From the comparison of both the charts, it can be seen that the proposed neutrosophic chart performs better and detects shifts earlier as compared to the classical counterpart.

#### Pakistan meteorological case-study

For the Meteorological Department dataset of Pakistan, the application of the proposed NCUSUM $\bar{X}$ chart is discussed. Since weather warning is a vital forecast because it is used for protecting people’s property and well-being as well as some socio-economic benefits. Thus, numerical weather experts use advanced mathematical formulas to predict the weather. Table 9 shows the weather forecast data information from 9 different cities in Pakistan for 12 months. The abbreviations symbols for the cities name are: Lahore = LHR, Karachi = KHI, Multan = MUX, Faisalabad = FSD, Islamabad = ISB, Peshawar = PEW, Hyderabad = HDD, Skardu = KDU and Gilgit = GIL.

**Table 9:** Meteorological department dataset for real-life example with design parameters *h* = 4.791, *H* = 1.409, *k* = 0.5 and *n* = 12.

	Weather Update by Month														
City	Jan	Feb	Mar	April	May	Jun	July	Aug.	Sep.	Oct	Nov	Dec	$\left[{\bar{X}}_{iL},{\bar{X}}_{iU}\right]$	$\left[M_{Li}^{+},M_{Ui}^{+}\right]$	$\left[M_{Li}^{-},M_{Ui}^{-}\right]$
LHR	[5.9,19.8]	[8.9,22.0]	[14.0,27.1]	[19.6,33.9]	[23.7,38.6]	[27.4,40.4]	[26.9,36.1]	[26.4,35.0]	[24.4,35.0]	[18.2,32.9]	[11.6,27.4]	[6.8,21.6]	[17.81,30.81]	[0.00,0.15]	[0.00,0.00]
KHI	[10.4,25.8]	[12.7,27.7]	[17.6,31.5]	[22.3,34.3]	[25.9,35.2]	[27.9,34.8]	[27.4,33.1]	[26.1,31.7]	[25.2,32.6]	[21,34.7]	[15.9,31.9]	[11.6,27.4]	[20.35,31.72]	[0.16,0.81]	[0.00,0.00]
MUX	[4.5,21]	[7.6,23.2]	[13.5,28.5]	[19.5,35.5]	[24.4,40.4]	[28.6,42.3]	[28.7,39.2]	[28,38]	[24.9,37.2]	[18.2,34.6]	[10.9,28.5]	[5.5,22.7]	[17.85,32.61]	[0.49,0.98]	[0.00,0.00]
FSD	[4.4,19.4]	[7.4,22.4]	[12.6,27.3]	[18.1,33.8]	[23.3,38.9]	[27.4,40.7]	[27.4,37.3]	[26.9,36.3]	[24.2,36]	[17.6,33.6]	[10.4,27.5]	[5.7,21.8]	[17.11,31.25]	[0.56,0.99]	[0.00,0.00]
ISB	[2.6,17.7]	[5.1,19.1]	[9.9,23.9]	[15,30.1]	[19.7,35.3]	[23.7,38.7]	[24.3,35]	[23.5,33.4]	[20.6,33.5]	[13.9,30.9]	[7.5,25.4]	[3.4,19.7]	[14.10,28.55]	[0.13,0.42]	[0.00,0.00]
PEW	[4,18.3]	[6.3,19.5]	[11.2,23.7]	[16.4,30]	[21.3,35.9]	[25.7,40.4]	[26.6,37.7]	[25.7,35.7]	[22.7,35]	[16.1,31.2]	[7.6,25.6]	[4.9,20.1]	[15.70,29.42]	[0.00,0.16]	[0.00,0.00]
HDD	[11.1,25]	[13.6,28.1]	[18.5,33.9]	[23,38.9]	[26.2,41.6]	[28.1,40.2]	[27.8,37.4]	[26.7,36.3]	[25.3,36.8]	[22.3,37.2]	[17.3,31.9]	[12.5,26.3]	[21.03,34.46]	[0.68,0.95]	[0.00,0.00]
KDU	[-12,-3]	[-11,-1]	[-5,5]	[0,12]	[3,15]	[7,20]	[10,23]	[10,22]	[5,20]	[-1,14]	[-6,7]	[-10,0]	[-0.83,11.16]	[0.00,0.00]	[-2.72,-2.52]
GIL	[-2.7,9.6]	[0.4,12.6]	[5.4,18.4]	[9.2,24.2]	[11.8,29]	[14.9,34.2]	[18.2,36.2]	[17.5,35.3]	[12.4,31.8]	[6.3,25.6]	[0.4,18.4]	[-2.3,11.6]	[7.62,23.91]	[0.00,0.00]	[-3.45,-3.02]

**Fig 5:**
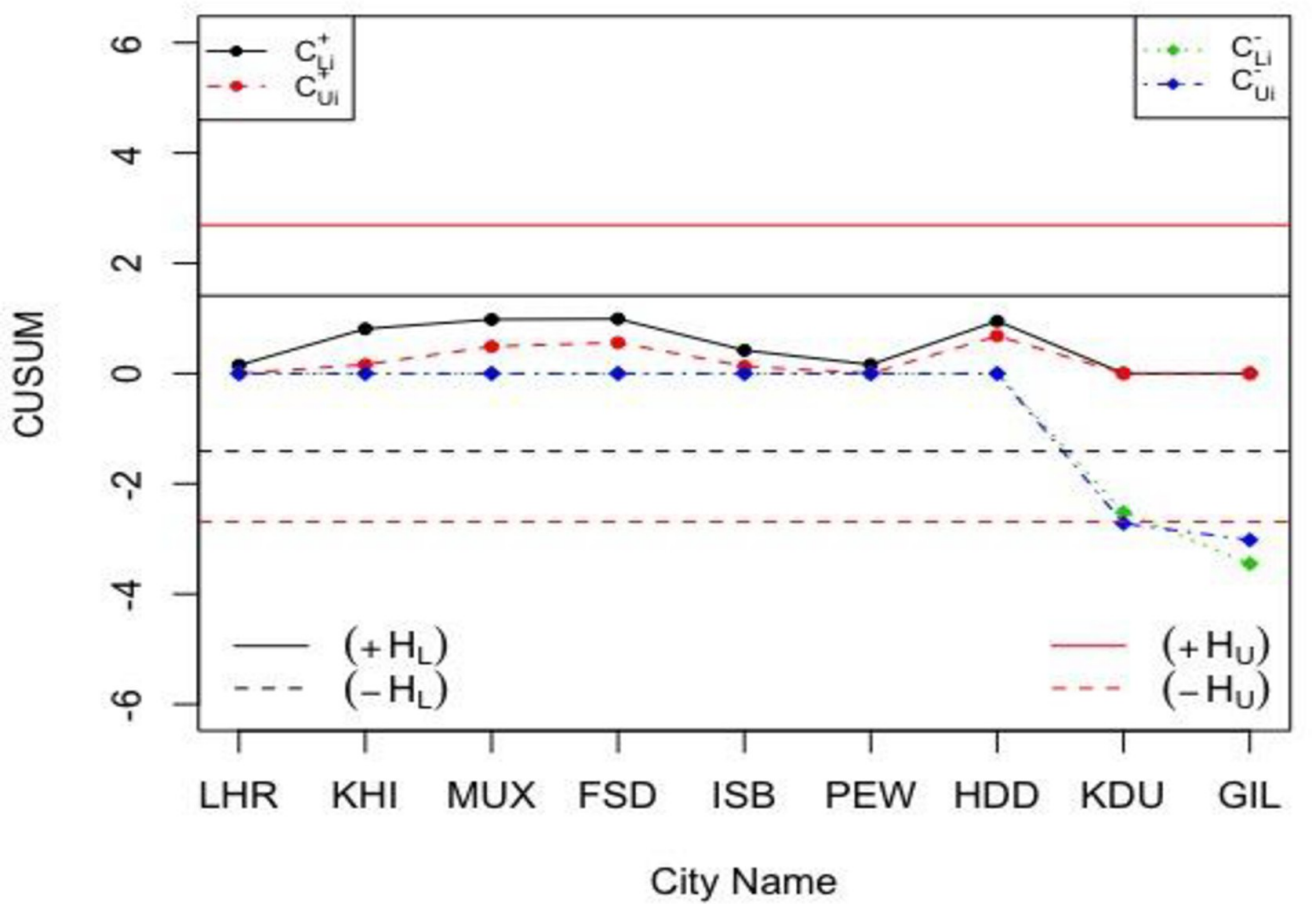
The proposed NCUSUM $\bar{X}$ chart for the weather condition in Pakistan with *H*_*N*_∈[1.4109, 2.6919] and *K*_*N*_∈[0.1472, 0.2809].

**Fig 6:**
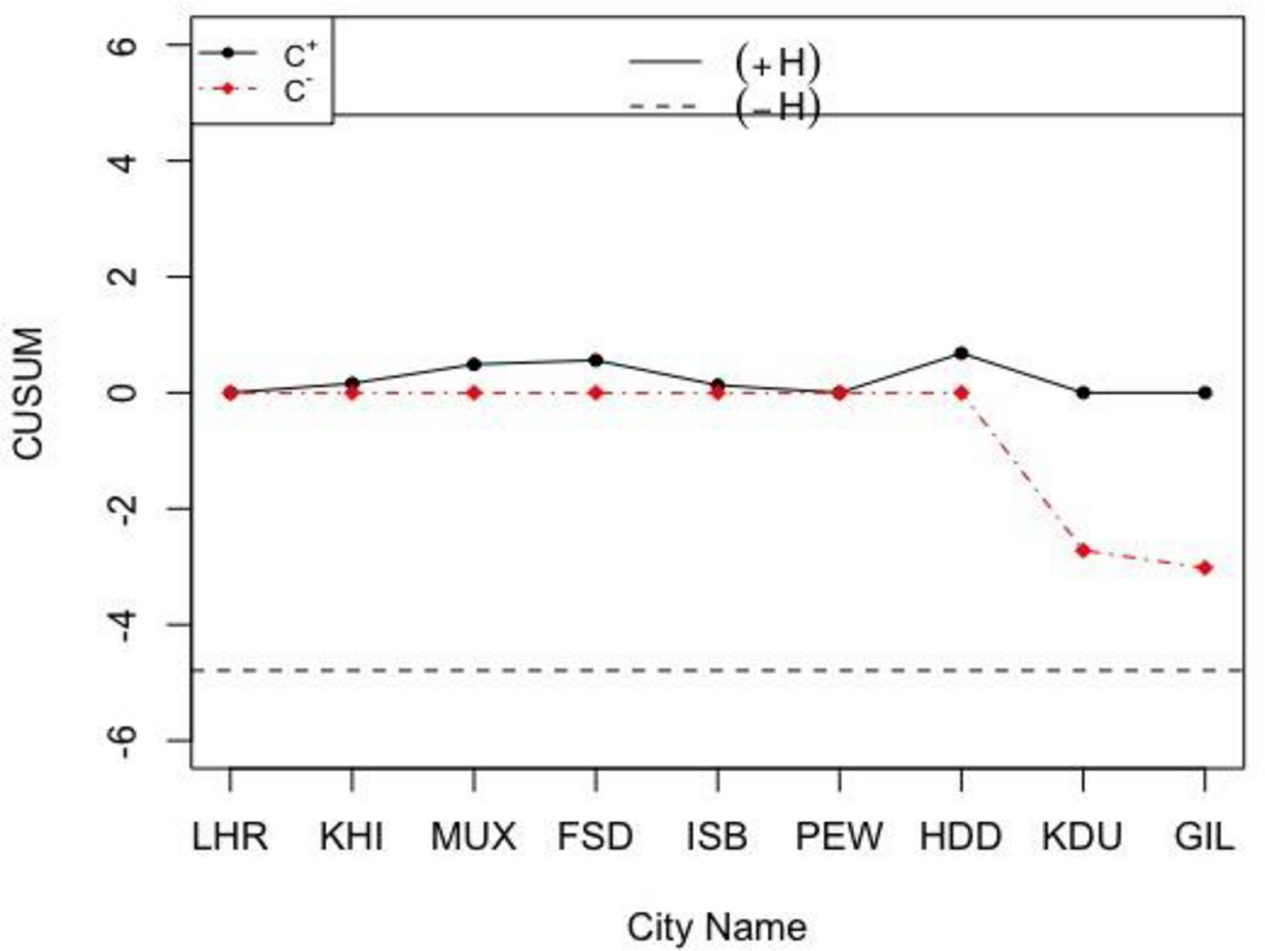
The classical CUSUM $\bar{X}$ chart for the weather data with *h* = 4.791 and *k* = 0.5.

Let the sample size and the in-control *ARL* values be *n*_*N*_ = 12 and *NARL*_0_ = 370, while the reference and decision interval values are *H*_*N*_∈[1.4109,2.6919] and *K*_*N*_∈[0.1472,0.2809] when *h* = 4.791, *k* = 0.5, and *σ*_0*N*_∈[1.0197,1.9464]. So, the calculated NCUSUM charting statistics (i.e. $M_{Ni}^{+}$ and $M_{Ni}^{-}$) are given in the last two columns of Table 9. The charting statistics of the proposed NCUSUM $\bar{X}$ chart are shown in Fig 5 and the ones of the classical CUSUM $\bar{X}$ chart are shown in Fig 6.

From Fig 5, the proposed NCUSUM $\bar{X}$ chart is found to be more efficient and gives better results compared to its classical counterpart in Fig 6; as it indicates downwards shift in the weather forecast and shows that the weather is very cold in Skardu and Gilgit cities. Note though, the classical CUSUM $\bar{X}$ chart reveals that all cities’ weather are within normal range and does not detect the shift at any point in the data. Thus, in comparison of both the control charts for monitoring weather forecasting, we can say that the NCUSUM $\bar{X}$ chart provides better prediction especially when meteorologists are not sure about the temperature values to be used for weather prediction. Therefore, the neutrosophic CUSUM $\bar{X}$ chart is recommended for weather prediction under the uncertainty environments.

## Conclusion and future recommendations

In this paper, a new NCUSUM $\bar{X}$ control chart is proposed. The neutrosophic run length performance of the proposed chart is computed using Monte Carlo simulations. The application of the proposed chart is illustrated using simulated data and two real-life examples using the PSO petroleum industry dataset and the weather forecasting dataset from nine cities in Pakistan. The results show that the NCUSUM $\bar{X}$ chart is better suited for use than the classical CUSUM $\bar{X}$ chart either when the data are inaccurate or when the operators do not have a complete dataset or for uncertain environments situations. The comparison study shows that the proposed neutrosophic chart is efficient in detecting small to large shifts in the process parameter than the classical chart. Note that the NCUSUM $\bar{X}$ chart is functional when the variable of interest adopts the neutrosophic normal distribution. The proposed neutrosophic chart is also recommended for monitoring the process mean shifts for the medical instruments, automobiles, aerospace and drinking water industries.

Further future works can be dedicated to the NCUSUM $\bar{X}$ chart that can be designed for non-normal distributions, as well as for the multiple dependent state sampling and repetitive sampling approaches.
